# Characterization and complete genome analysis of the surfactin-producing, plant-protecting bacterium *Bacillus velezensis* 9D-6

**DOI:** 10.1186/s12866-018-1380-8

**Published:** 2019-01-08

**Authors:** Elliot Nicholas Grady, Jacqueline MacDonald, Margaret T. Ho, Brian Weselowski, Tim McDowell, Ori Solomon, Justin Renaud, Ze-Chun Yuan

**Affiliations:** 1 Department of Microbiology & Immunology, Schulich School of Medicine & Dentistry, Dental Science Building Rm. 3014, University of Western Ontario, London, ON N6A 5C1 Canada; 2 London Research and Development Centre, Agriculture & Agri-Food Canada, 1391 Sandford Street, London, ON N5V 4T3 Canada

**Keywords:** Antibacterial, Antifungal, Genome sequencing, Induced systemic resistance (ISR), Plant-microbe interactions, Secondary metabolites

## Abstract

**Background:**

*Bacillus velezensis* is an endospore-forming, free-living soil bacterium with potential as a biopesticide against a broad spectrum of microbial pathogens of plants. Its potential for commercial development is enhanced by rapid replication and resistance to adverse environmental conditions, typical of *Bacillus* species. However, the use of beneficial microbes against phytopathogens has not gained dominance due to limitations that may be overcome with new biopesticidal strains and/or new biological knowledge.

**Results:**

Here, we isolated *B. velezensis* strain 9D-6 and showed that it inhibits the in vitro growth of prokaryotic and eukaryotic pathogens, including the bacteria *Bacillus cereus*, *Clavibacter michiganensis*, *Pantoea agglomerans*, *Ralstonia solanacearum*, *Xanthomonas campestris*, and *Xanthomonas euvesicatoria*; and the fungi *Alternaria solani*, *Cochliobolus carbonum*, *Fusarium oxysporum*, *Fusarium solani*, *Gibberella pulicaris*, *Gibberella zeae*, *Monilinia fructicola*, *Pyrenochaeta terrestris* and *Rhizoctonia solani*. Antimicrobial compounds with activity against *Clavibacter michiganensis* were isolated from *B. velezensis* 9D-6 and characterized by high resolution LC-MS/MS, yielding formulae of C_52_H_91_N_7_O_13_ and C_53_H_93_N_7_O_13_, which correspond to [Leu^7^] surfactins C_14_ and C_15_ (also called surfactin B and surfactin C), respectively. We further sequenced the *B. velezensis* 9D-6 genome which consists of a single circular chromosome and revealed 13 gene clusters expected to participate in antimicrobial metabolite production, including surfactin and two metabolites that have not typically been found in this species - ladderane and lantipeptide. Despite being unable to inhibit the growth of *Pseudomonas syringae* DC3000 in an in vitro plate assay, *B. velezensis* 9D-6 significantly reduced root colonization by DC3000, suggesting that 9D-6 uses methods other than antimicrobials to control phytopathogens in the environment. Finally, using in silico DNA-DNA hybridization (*is*DDH), we confirm previous findings that many strains currently classified as *B. amyloliquefaciens* are actually *B. velezensis*.

**Conclusions:**

The data presented here suggest *B. velezensis* 9D-6 as a candidate plant growth promoting bacterium (PGPB) and biopesticide, which uses a unique complement of antimicrobials, as well as other mechanisms, to protect plants against phytopathogens. Our results may contribute to future utilization of this strain, and will contribute to a knowledge base that will help to advance the field of microbial biocontrol.

**Electronic supplementary material:**

The online version of this article (10.1186/s12866-018-1380-8) contains supplementary material, which is available to authorized users.

## Background

Pathogens of plants are a major constraint to global food production, both in the field and as post-harvest diseases. While synthetic pesticides, including bactericides and fungicides, can be effective, the reliance on synthetic inputs in modern agriculture can cause serious environmental problems by affecting soil fertility, the development of insect resistance, and bioaccumulation of toxic residues in wildlife, livestock, and humans [1]. Such concerns have prompted research into alternative, sustainable strategies to manage plant pests and diseases.

The millions of microbes that live in soil represent a rich source of biodiversity with great potential for the development of biopesticides. Since biopesticides are derived from natural microorganisms, they are often less toxic and affect fewer non-target organisms compared with synthetic pesticides. In addition, they can be effective in small quantities and are biodegradable, largely avoiding pollution problems. Biopesticides are promising in promoting agricultural sustainability and intensification, helping to meet today’s complex challenges.

Numerous microorganisms have been successfully developed into biopesticides at the commercial level, most notably strains of *Bacillus thuringiensis* (Bt) against certain types of insects. However, the use of beneficial microbes has not gained dominance or popularity for control of microbial pathogens, which may partially be due to the inconsistent responses among plant cultivars and field sites [2]. These limitations may be overcome by developing new biopesticidal strains and further research into the biology of plant-microbe and microbe-microbe interactions.

Many of the bacterial antagonists to microbial phytopathogens also belong to the genus *Bacillus* [3], and the use and number of antagonistically important *Bacillus* species is increasing very rapidly. *Bacillus* species synthesize various types of lipopeptide secondary metabolites with specific activities against plant pathogens, including many potent amphiphilic and surfactant lipopeptides comprising bacillomycins, iturins, surfactins, and mycosubtilin [4]. In addition to their broad spectrum of biocontrol ability, *Bacillus* species replicate rapidly and are resistant to adverse environmental conditions [5].

*Bacillus velezensis* was originally described in 2005 [6], and various strains have since been researched for their potential as biopesticides [7, 8]. Here we isolated and characterized *B. velezensis* strain 9D-6 from a farm in Southern Ontario, Canada. This bacterial strain exhibits potent biocontrol activity against a wide range of bacterial and fungal pathogens. We identified antimicrobial compounds produced by *B. velezensis* 9D-6 as bacterial cyclic lipopeptides [Leu^7^] surfactin C_14_ and [Leu^7^] surfactin C_15_. We further describe additional plant protection mechanisms of *B. velezensis* 9D-6 as it reduced root colonization by the plant pathogen *Pseudomonas syringae* DC3000. Moreover, we sequenced the genome of *B. velezensis* 9D-6 and the complete genome sequence presented here will facilitate future research and development of this and related organisms in agriculture and the biotechnology industry. Overall, the results indicate the potential of developing *B. velezensis* 9D-6 as a biopesticides for sustainable agriculture.

## Results

### Isolation of bacteria with antimicrobial activities

**Fig. 1:**
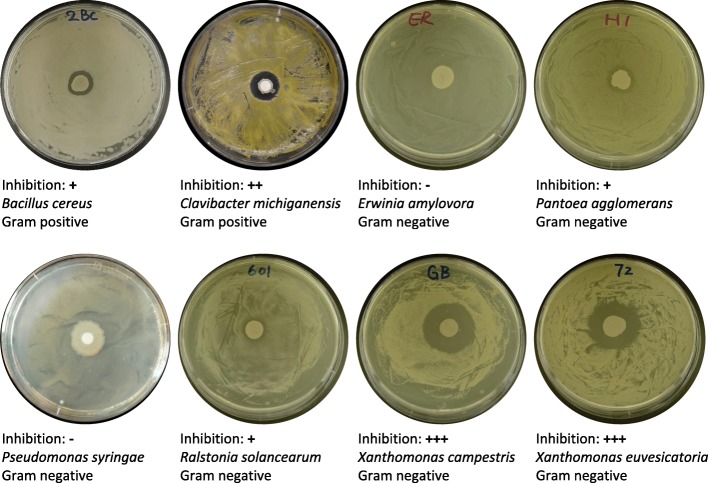
Inhibition of bacterial pathogens by *B. velezensis* 9D-6 in in vitro plate assays. Representative plates are shown, along with average degree of inhibition zone of clearance measurements: (−) = no inhibition, (+) = 0.1–1.9 mm, (++) = 2.0–4.9 mm, (+++) = 5.0–9.9 mm, (++++) 10.0 mm+. *B. cereus* causes necrotic enteritis of mammals, including humans [52]; *C. michiganensis* causes bacterial canker of tomato [10]; *E. amylovora* causes fire blight of apple and pear [53]; *P. agglomerans* causes several diseases of several crops [54]; *P. syringae* DC3000 causes bacterial speck of tomato, Arabidopsis, and *Nicotiana benthamiana* [55]; *R. solanacearum* causes several diseases of several crops [56]; *X. campestris* causes black rot of the Brassicaceae family (including broccoli and radish) [57]; *X. euvesicatoria* causes bacterial spot of tomato [58]

**Fig. 2:**
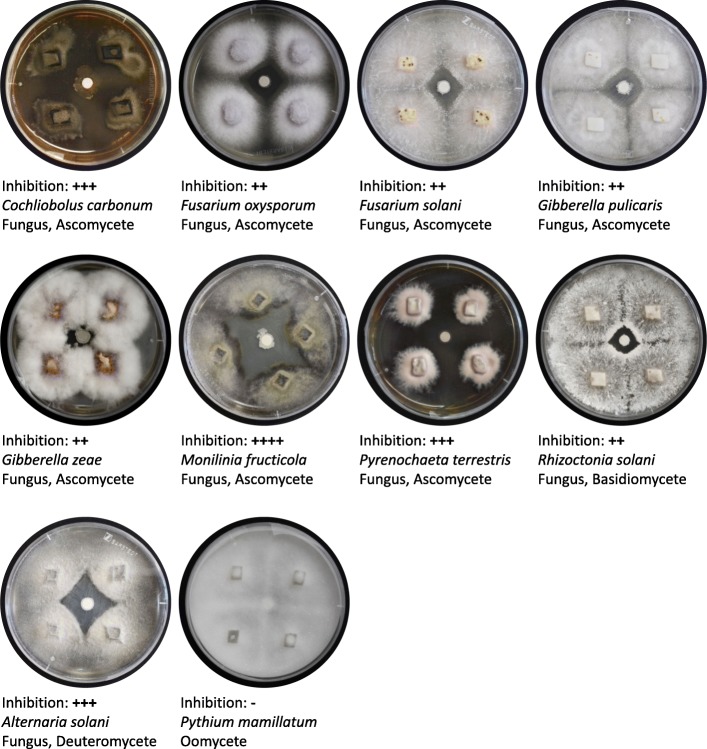
Inhibition of eukaryotic pathogens by *B. velezensis* 9D-6 in in vitro plate assays. Representative plates are shown, along with average degree of inhibition zone of clearance measurements: (−) = no inhibition, (+) = 0.1–1.9 mm, (++) = 2.0–4.9 mm, (+++) = 5.0–9.9 mm, (++++) 10.0 mm+. *C. carbonum* causes Northern leaf spot and ear rot of sorghum, corn, apple, and peach [59]; *F. oxysporum* causes wilt and root rot of several crops [60]; *F. solani* causes mycotic keratitis of mammals (including humans) and root rot of plants [61]; *G. pulicaris* causes potato dry rot [62]; *G. zeae* causes wheat head blight and maize ear rot [63]; *M. fructicola* causes brown rot of nectarine and other stone fruit [64]; *P. terrestris* causes pink rot of onion [65]; *R. solani* causes several diseases of several crops [66]; *A. solani* causes early blight of tomato and potato [67]; *P. mamillatum* causes damping off and root rot of sevral crops [68, 69]

Out of six hundred bacterial isolates from a remediated potato rhizosphere in Norfolk County, Ontario, Canada, *isolate* 9D-6 exhibited the highest degree of microbial growth inhibition. Zones of microbial inhibition on solid medium around discs inoculated with 9D-6 indicated that it inhibits growth of the Gram positive bacteria *Bacillus cereus* and *Clavibacter michiganensis*; and of the Gram negative bacteria *Pantoea agglomerans*, *Ralstonia solanacearum*, *Xanthomonas campestris*, and *Xanthomonas euvesicatoria*; but not *Erwinia amylovora* or *Pseudomonas syringae* DC3000. It inhibited growth of the ascomycete fungi *Cochliobolus carbonum*, *Fusarium oxysporum*, *Fusarium solani*, *Gibberella pulicaris*, *Gibberella zeae*, *Monilinia fructicola*, and *Pyrenochaeta terrestris*; the basidiomycete fungus *Rhizoctonia solani*; and the deuteromycete fungus *Alternaria solani*; but not the oomycete *Pythium mamillatum*. In summary, isolate 9D-6 inhibited growth of six of the seven bacterial pathogens and all nine of the fungal pathogens that were tested (Figs. 1 and 2).

We further tested growth of strain 9D-6 at various temperatures and pH. Strain 9D-6 grew in liquid LB medium at a temperature range of at least between 12 °C and 50 °C, and at a pH between 5 and 8, with optimal growth around 30 °C and pH 7 (Additional files 1 and 2).

### *B. velezensis* 9D-6 phylogeny

BLASTn analysis of 16S rDNA amplified from isolate 9D-6 returned a > 99% identity match with the reference strain *B. velezensis* G341. Therefore, isolate 9D-6 is named *B. velezensis* 9D-6.

**Fig. 3:**
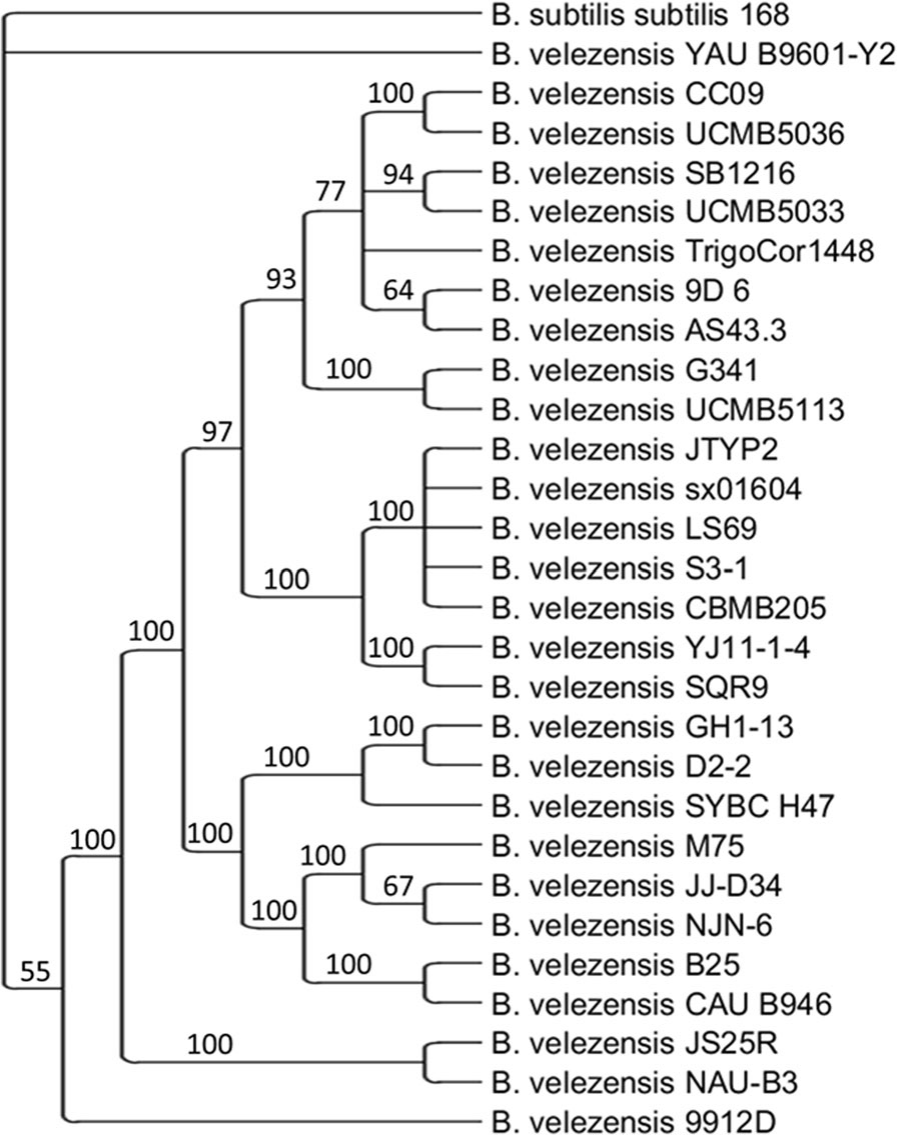
Majority-rule consensus tree of *rpo* genes rooted with *B. subtilis subtilis* 168 as the outgroup. Bayesian clade credibility values are indicated at nodes. GenBank acession numbers are listed in brackets: *B. subtilis subtilis* strain 168 (CP01 0052), B. velezensis strains 9912D (CP017775), 9D-6 (CP020805), AS43.3 (CP003838), B25 (LN999829), CAU B946 (HE617159), CBMB205 (CP011937 and CP014838), CC09 (CP015443), D2–2 (CP014990), G341 (CP011686), GH1–13(CP019040), JJ-D34 (CP011346), JS25R (CP009679), JTYP2 (CP020375), LS69 (CP015911), M75 (CP016395), NAU-B3 (HG514499), NJN-6 (CP007165), S3–1 (CP016371), SB1216 (CP015417), SQR9 (CP006890), SYBC H47 (NZ_CP017747), sx01604 (CP018007), TrigoCor1448 (CP007244), UCMB5033 (HG328253), UCMB5036 (HF563562), UCMB5113 (HG328254), YAU B9601-Y2 (HE774679), YJ11–1-4 (CP011347)

To estimate the evolutionary position of *B. velezensis* 9D-6 relative to 27 other *B. velezensis* strains for which complete genomes are available, a phylogenetic tree was constructed based on the RNA polymerase β subunit (*rpoB*) gene. Compared with the *16S rRNA* gene, *rpoB* provides better resolution between closely related organisms [9]. The phylogenetic analysis suggests that *B. velezensis* 9D-6 is most closely related to *B. velezensis* AS43.3, and is also closely related to *B. velezensis* strains CC09, SB1216, TrigoCor1448, UCMB5033, and UCMB5036, followed together by UCMB5113 and the reference strain G341 (Fig. 3).

In silico DNA-DNA hybridization (*is*DDH) was performed to confirm 9D-6 as a member of *B. velezensis* and to validate its position in the phylogenetic tree. Of the strains included in the tree (Fig. 3), the highest *is*DDH values with the genome of *B. velezensis 9D-6 were obtained for strains TrigoCor1448 (92.5%), AS43.3 (92.0%), UCMB5113 (91.7%), G341 (91.7%), UCMB5033 (91.2%), SB1216 (91.1%), UCMB5036 (89.3%), and CC09 (89.1%). These eight strains form a cluster with B. velezensis* 9D-6 in the *rpoB* phylogenetic tree. The values for *is*DDH between *B. velezensis* 9D-6 and that of the other *B. velezensis* strains range from 79 to 86%. In contrast, the value for *is*DDH is 21% between *B. velezensis* 9D-6 and *B. subtilis subtilis* 168. However, it ranges between 55 and 92% between *B. velezensis* 9D-6 and 22 *B. amyloliquefaciens* strains with complete genomes: 15 of these values are between 79 and 92%, while the remaining six (including the type strain DSM 7) are between 55 and 56%.

### *B. velezensis* 9D-6 produces antimicrobial compounds [Leu^7^] surfactin C_14_ and [Leu^7^] surfactin C_15_

**Fig. 4:**
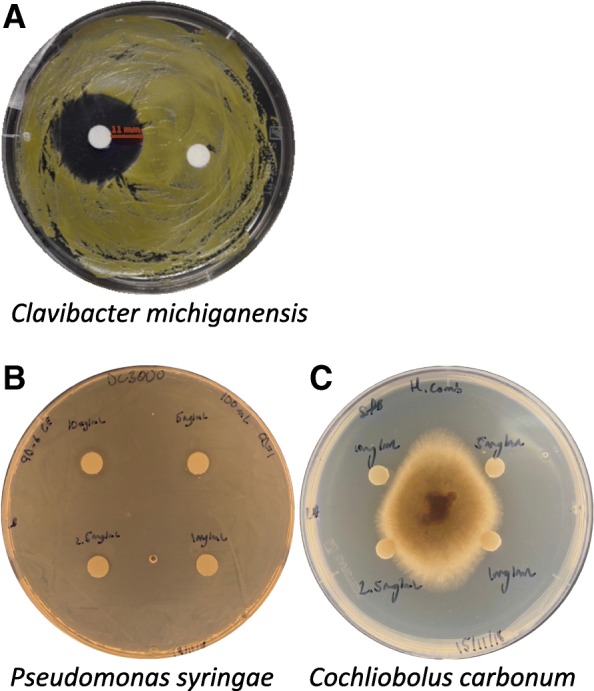
Metabolite fraction from *B. velezensis* liquid culture demonstrates antimicrobial activity. **a** The antimicrobial disc (left) was prepared by adding 50 μL of fraction to filter paper, and placed onto an LB agar plate spread with *C. michiganensis*. The control disc (right) used acetonitrile in place of the metabolite fraction. **b** Antimicrobial discs with surfactin concentrations between 1 mg/mL and 10 mg/mL were placed onto an LB agar plate spread with *P. syringae* DC3000*.* **c** Antimicrobial discs with surfactin concentrations between 1 mg/mL and 10 mg/mL were placed onto an LB agar containing a central agar plug of *C. carbonum*

To identify the antimicrobial compounds that contribute to biocontrol, *B. velezensis* 9D-6 metabolites were isolated into fractions using liquid chromatography, and each fraction was tested for antibacterial activity against the Gram-positive bacterium *C. michiganensis*, chosen due to its rapid growth rate and agricultural relevance [10, 11]. A fraction demonstrating activity (Fig. 4a) was then subjected to LC-MS to identify the active compound.

**Fig. 5:**
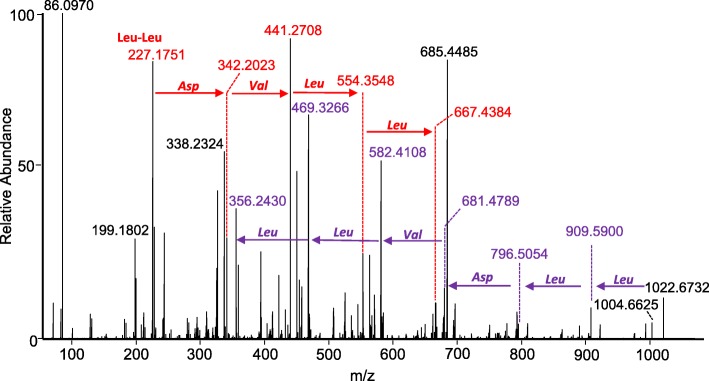
MS/MS de novo sequencing identified m/z 1022.6732 (C_53_H_93_N_7_O_13_) as [Leu^7^] surfactin C_15_. Purple arrows show changes in fragment m/z due to loss of the indicated amino acids from the full-length molecule. Red arrows show changes in fragment m/z due to addition of the indicated amino acids, starting from the di-leucine fragment at m/z 227.1751

The two major peaks observed had predicted chemical formulas of C_52_H_91_N_7_O_13_, and C_53_H_93_N_7_O_13_. Antibase identified the lipopeptide surfactins as possible identifications. Comparison of the MS/MS of compound C_52_H_91_N_7_O_13_ with previously published MS/MS spectra [12] identified this peak as [Leu^7^] surfactin C_14_ (sometimes called surfactin B). MS/MS of the peak C_53_H_93_N_7_O_13_ allowed partial amino acid sequence to be determined by de novo sequencing (Fig. 5), suggesting it is [Leu^7^] surfactin C_15_ (sometimes called surfactin C).

The surfactin fraction was then tested against the Gram-negative bacterium *P. syringae* DC3000 and the ascomycete fungus *C. carbonum* for verification (Fig. 4b-c). The antimicrobial disc inhibited *C. carbonum* when inoculated with surfactin concentrations between 1 mg/mL and 10 mg/mL. However, for *P. syringae* DC3000, the zones of inhibition were only apparent at the higher concentrations (5 mg/mL and 10 mg/mL) and were not very large, consistent with the inability of live *B. velezensis* to antagonize this bacterium (Fig. 1).

### *B. velezensis* 9D-6 reduces the attachment and infection of Arabidopsis by ***P. syringae*** DC3000

To determine whether *B. velezensis* 9D-6 is able to inhibit microbial phytopathogenesis by mechanisms other than antimicrobial production, we tested its ability to reduce root colonization by *P. syringae* DC3000, a Gram-negative bacterium. Since growth of *P. syringae* DC3000 was not inhibited by *B. velezensis* 9D-6 in the in vitro plate assay (Fig. 1), reduced root colonization in response to *B. velezensis* 9D-6 should be due to a mechanism other than production of bacteriocidal or bacteriostatic antimicrobials.

**Fig. 6:**
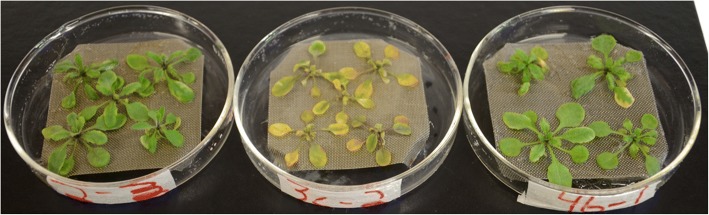
Effect of *B. velezensis* 9D-6 co-inoculation with *P. syringae* DC3000 on *A. thaliana* disease symptoms. *A. thaliana* seedlings were cultured in hydroponic medium for seven days with *B. velezensis* 9D-6 alone (left), *P. syringae* DC3000 alone (middle), or both *B. velezensis* 9D-6 and *P. syringae* DC3000 (right). Representative samples are shown

**Fig. 7:**
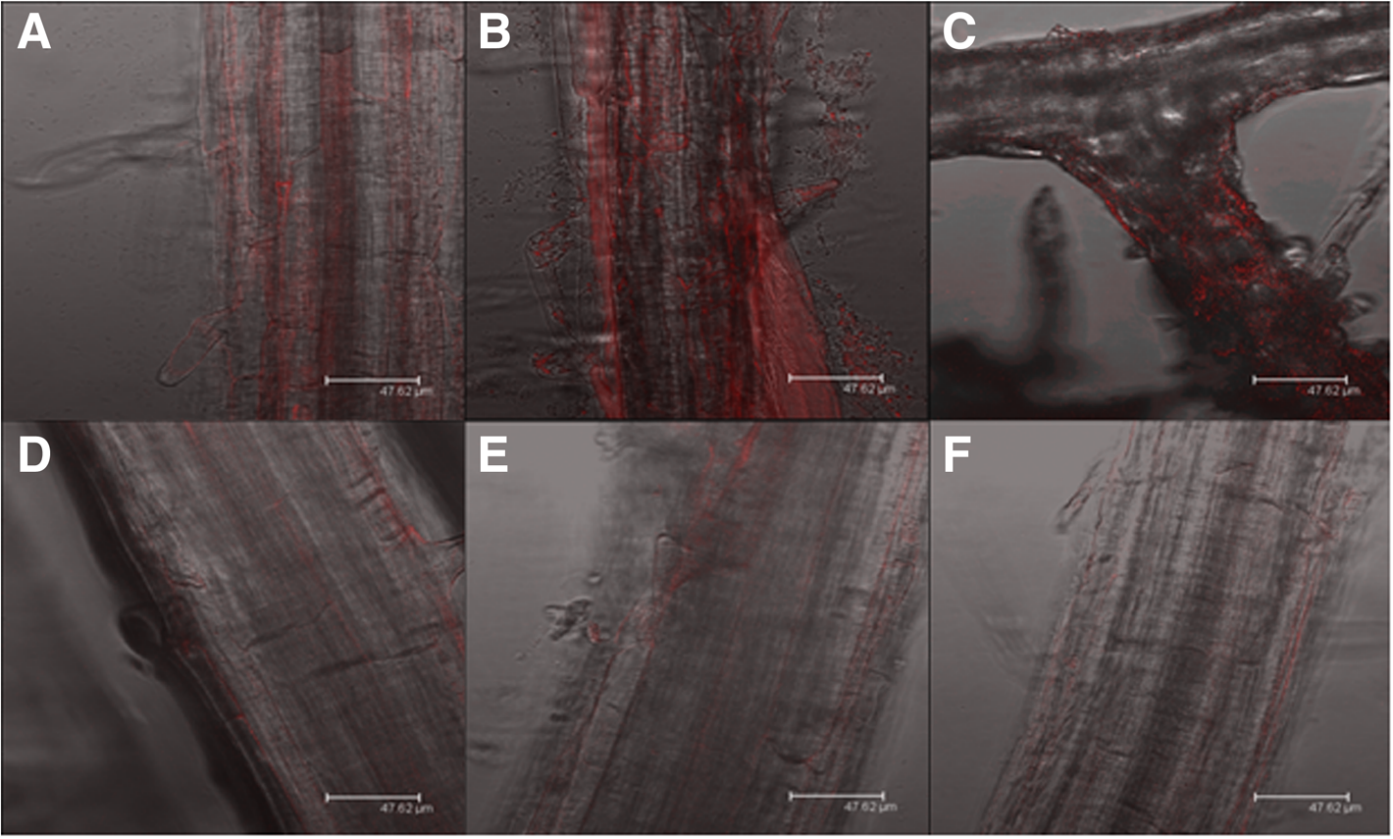
Effect of *B. velezensis* 9D-6 co-inoculation on *P. syringae* DC3000 root colonization. *A. thaliana* seedlings were cultured in hydroponic medium for seven days with RFP-labeled *P. syringae* DC3000 alone at 2.5 × 10^5^ CFU/mL, 2.5 × 104 CFU/mL, or 2.5 × 10^3^ CFU/mL (A-C respectively), or with both *B. velezensis* 9D-6 at 2.5 × 10^5^ CFU/mL and *P. syringae* DC3000 at 2.5 × 10^5^ CFU/mL, 2.5 × 10^4^ CFU/mL, or 2.5 × 10^3^ CFU/mL (D-F respectively). Roots were imaged using confocal microscopy to detect red fluorescence indicative of *P. syringae* DC3000 attachment. Representative samples are shown

Co-inoculation of *A. thaliana* seedlings with *B. velezensis* 9D-6 and *P. syringae* DC3000 produced milder disease symptoms compared to inoculation with *P. syringae* DC3000 alone. By contrast, *B. velezensis* 9D-6 alone produced no visible symptoms of disease (Fig. 6). Co-inoculation of *A. thaliana* seedlings with *B. velezensis* 9D-6 and RFP-labeled *P. syringae* DC3000 also resulted in a notable decrease in root colonization by the pathogen at all tested concentrations, as indicated by an observable decrease in RFP fluorescence (Fig. 7).

### The *B. velezensis* 9D-6 genome sequencing and analysis

To allow further investigation into the biocontrol mechanisms of *B. velezensis* 9D-6 and its application for sustainable agriculture, we determined its complete genome.

**Fig. 8:**
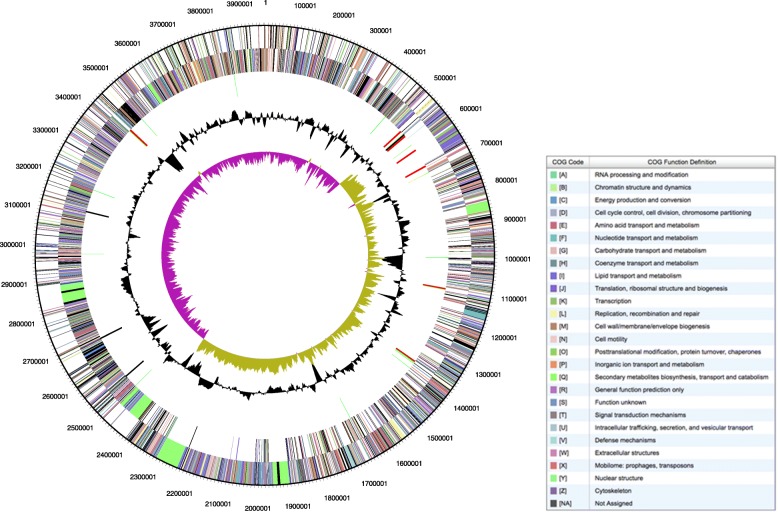
Visual representation of the *B. velezensis* 9D-6 genome From outer to inner rings: forward coding DNA sequence genes coded to COG functions, reverse coding DNA sequence genes coded to COG functions, RNAs (tRNAs in green, rRNAs in red, and other RNAs in black), GC content (black), and GC skew (magenta and yellow). Map was generated from the Joint Genome’s Institute (JGI) Integrated Microbial Genomics (IMG) Database [48]

The *B. velezensis* 9D-6 genome consists of a single 3.96 Mb circular chromosome (Fig. 8), which fits within the range of 3.81 to 4.24 Mb reported for other completed genomes of the species. Like nearly 80% of those strains, it does not harbor a plasmid. The *B. velezensis* 9D-6 genome is predicted to include 3942 total genes, of which 3849 (97.6%) are protein coding genes, 93 (2.4%) are RNA coding, and 82 (2.1%) are pseudogenes (Additional file 3). Of the RNA coding genes, 21 are predicted to code rRNA and 68 are predicted to encode tRNA. Among the predicted genes, 2736 (69.4%) are associated with general COG function categories. The distribution of genes in these categories is presented in Additional file 4.

A core genome containing 2574 coding sequences is shared among *B. velezensis*, the closely related species *Bacillus amyloliquefaciens*, and *B. subtilis subtilis* 168 [13]. Among *B. velezensis* strains with completed genomes, the number of coding sequences is typically between 3600 and 4000 (with a clear outlier of 4411 for *B. velezensis* SRCM101413) and the number for *B. velezensis* 9D-6 fits within this expected range. Similarly, the 46.4% GC content of *B. velezensis* 9D-6 is comparable to the other completed *B. velezensis* genomes, which range from 45.9 to 46.8% GC. The 9D-6 genome sequence was deposited in GenBank (CP020805): https://www.ncbi.nlm.nih.gov/nuccore/CP020805

### Special genomic features

The *B. velezensis* 9D-6 genome is predicted to contain thirteen gene clusters involved in antimicrobial production (Table 1), most of which are conserved in all *B. velezensis* strains (bacilysin, surfactin, macrolactin, fengycin, bacillaene, difficidin, bacillibactin [7, 13], butirosin, and a terpene [13]). AntiSMASH analysis of the *B. velezensis* 9D-6 genomic surfactin cluster predicted a lipopeptide sequence of ELLVDLL, consistent with the peptide ring of surfactin determined by the LC-MS/MS and de novo peptide sequencing. In contrast, the clusters predicted to produce ladderane and lantipeptide have not typically been found in this species. The lantipeptide cluster contains a predicted set of ten core biosynthetic genes, including the crucial modification enzyme LanM, but does not contain obvious hits for some other components including LanA and LanT. The ladderane cluster contains four core biosynthetic genes, encoding an acyl carrier protein, beta-ketoacyl synthase, 3-oxoacyl-ACP synthase, and 3-oxoacyl-ACP reductase.

**Table 1:** Antimicrobial gene clusters present in *B. velezensis* 9D-6

Predicted product	Enzyme complex	Genome location
Bacilysin		174,243–215,661
Surfactin (cyclic lipopeptide)	nrps	820,469–885,876
Ladderane		1,177,384–1,218,583
Butirosin	t2pks	1,451,008–1,492,252
Terpene		1,575,053–1,595,793
Lantipeptide		1,714,976–1,743,864
Macrolactin (polyketide)	transatpks	1,893,381–1,979,274
Bacillaene (polyketide)	transatpks; nrps	2,209,276–2,311,962
Fengycin (bacteriocin)	transatpks; nrps	2,364,456–2,489,567
Terpene		2,517,434–2,539,317
Unknown	t3pks	2,602,290–2,643,390
Difficidin (polyketide)	transatpks	2,801,002–2,901,455
Bacillibactin (bacteriocin)	nrps	3,562,018–3,628,825

To determine the prevalence of the lantipeptide and ladderane clusters among strains of *B. velezensis*, we search the 27 other strains that are represented in our phylogenetic tree, all of which have completed genomes. Clusters for lantipeptides were found in 13 of the 27 other strains, while clusters for ladderane were found in only 2 of the 27 other strains. One strain other than 9D-6 harbors clusters for both lantipeptide and ladderane (strain UCMB5113). Of interest, other uncommon clusters among the strains are for thiopeptide (in 4 strains: D2–2, M75, JJ-D34, and B25) and phosphonate (in 3 strains: YAU B9601-Y2, JS25R, and NAU-B3) (Table 2). Consistent with previous findings, little correlation was observed between the presence of any specific cluster and its phylogenetic position within the species. *B. velezensis* SRCM101413, the strain with the largest number of coding sequences, contains a cluster for ladderane, but not for lantipeptide, thiopeptide, nor phosphonate.

**Table 2:** Less common antimicrobial gene clusters present in *B. velezensis* strains

Strain	Ladderane	Lantipeptide	Thiopeptide	Phosphonate
YAU B9601-Y2		✓		✓
CC09				
UCMB5036				
SB1216				
UCMB5033				
TrigoCor1448				
9D-6	✓	✓		
AS43.3	✓			
G341		✓		
UCMB5113	✓	✓		
JTYP2		✓		
sx01604		✓		
LS69		✓		
S3–1		✓		
CBMB205		✓		
YJ11–14				
SQR9				
GH1–13		✓		
D2–2			✓	
SYBC H47				
M75		✓	✓	
JJ-D34				
NJN-6		✓		
B25		✓	✓	
CAU B946				
JS25R				✓
NAU-B3				✓
9912D		✓		

Manual mining of *B. velezensis* 9D-6 also identified a gene for butanediol dehydrogenase and genes predicted to encode components of secretion systems including the inner-membrane spanning twin-arginine translocation (Tat) system, SecYEG translocon, and the Type VII/ESX secretion system.

## Discussion

The isolate 9D-6 was named *B. velezensis* 9D-6 based on a > 99% 16S rDNA identity match with the reference strain *B. velezensis* G341. In silico DNA-DNA hybridization (*is*DDH) also confirmed 9D-6 as a member of *B. velezensis*, with the *is*DDH value between *B. velezensis* 9D-6 and other *B. velezensis* strains ranging from 79 to 92%. This is above the typical standard of 70% for delineating species. Comparing *B. velezensis* 9D-6 to members of the related *B. amyloliquefaciens* gave *is*DDH values between 55 and 92%, with no values between 57 and 78%, in support of previous findings that many strains currently classified as *B. amyloliquefaciens* are actually *B. velezensis* [14].

A key distinguishing feature among strains of *B. velezensis* is the complement of secondary metabolite clusters that are predicted contributors to antimicrobial activities [7]. While many such clusters are common to multiple species of *Bacillus*, others are specific to certain strains of *B. velezensis*, with little correlation to the predicted phylogenetic relationships among these strains [7, 13]. Such ancillary clusters may function primarily to control other *Bacillus* species and Gram-positive bacteria [7].

The *B. velezensis* 9D-6 genome is predicted to contain thirteen antimicrobial gene clusters. While the majority of the thirteen are conserved in all *B. velezensis* strains, the clusters predicted to produce ladderane and lantipeptide have not typically been found in this species. The cluster for ladderane has previously been found in *Bacillus* strains isolated from marine sponges [15], while various *Bacillus* strains produce lantipeptides [16].

Lantipeptides are ribosomally synthesized peptides that are extensively post-translationally modified, and often disrupt the integrity of Gram-positive bacterial cell walls. For a class II lantipeptide, which was predicted for *B. velezensis* 9D-6, the ribosomally produced precursor peptide, LanA, is processed by two conserved enzymes, LanM and LanT. LanM acts via an N-terminal dehydratase domain and a C-terminal cyclase domain, whereas LanT performs cleavage of the leader peptide. Additional post-translational modifications can be performed by other enzymes, leading to further structural diversity among lantipeptides [17]. While the *B. velezensis* 9D-6 cluster contains a gene for LanM, obvious matches for LanA and LanT appear to be absent, raising the possibility of a pseudogene cluster or misidentification.

Ladderanes are a type of lipid known to be present in membranes that surround anammoxosomes, which are involved in anaerobic ammonium oxidation by bacteria in the phylum Planctomyces. While gene clusters for ladderane biosynthesis have been identified, the precise biosynthetic pathway is still unknown [18] leaving a greater possibility for misidentification of this cluster. In fact, a large overlap has been found between gene clusters for ladderane synthesis and those for synthesis of aryl polyenes [19], which offer protection from reactive oxygen species [20]. Aryl polyene gene clusters have been found mainly in Gram-negative bacteria, many of which are either commensals or pathogens of eukaryotes. Their phylogenetic distribution has been described as markedly discontinuous, with clusters present in some strains but not others of most genera [19]. This last point is consistent with our finding of the ladderane gene cluster in *B. velezensis* 9D-6 but not in most of the other *B. velezensis* strains.

The genome of *B. velezensis* 9D-6 also encodes a putative butanediol dehydrogenase, which is a critical enzyme in the production of butanediol, known to induced systemic resistance (ISR) in plants [21]. The presence of this gene therefore suggests that *B. velezensis* 9D-6 may contribute to plant defenses via ISR, whereby plants are primed for faster and stronger defenses against pathogens.

Additionally present are genes predicted to encode components of secretion systems, which may help mediate relationships between *B. velezensis* 9D-6 and other organisms, including plants and pathogenic microbes. Among these are components of the inner-membrane spanning twin-arginine translocation (Tat) system and SecYEG translocon, and the Type VII/ESX secretion system, whose function remains unknown. The twin-arginine translocation (Tat) system transports fully folded proteins to the cell envelope or extracellular space [22], while the SecYEG translocon participates in the insertion of membrane proteins [23].

*B. velezensis* 9D-6 inhibited in vitro growth of six of the seven bacterial pathogens and all nine of the fungal pathogens that were tested. This activity was shown to be due in part to the active compounds [Leu^7^] surfactin C_14_ (sometimes called surfactin B) and [Leu^7^] surfactin C_15_ (sometimes called surfactin C). These surfactins were shown to be inhibitory to the bacterial phytopathogen *C. michiganensis* and the ascomycete fungus *C. carbonum*, but not to *P. syringae* DC3000, consistent with the inability of live *B. velezensis* to antagonize this latter bacterium. Since not all test organisms used for the live *B. velezensis* disc assays were tested with surfactin, this antimicrobial fraction does not necessarily contribute to the activity against all of these organisms.

Surfactins are cyclic lipopeptides, each comprised of seven amino acids and a hydrophobic fatty acid chain that is at least thirteen carbons long. They are synthesized independently of messenger RNA by nonribosomal peptide synthetases (NRPSs), which are multienzyme complexes that can incorporate a mixture of D- and L-amino acids. While structures have been identified that vary in amino acid identity at the 7th position, those that retain the ELLVDLL sequence have variously been called [Leu]- or [Leu^7^] surfactin [24, 25], surfactin A (where isoforms with V and I at position 7 are called surfactin B and C, respectively [26, 27], or simply surfactin; while the nature of the acyl chain is designated as C_13_, C_14_, C_15,_ etc. (sometimes called surfactin A, B, C, etc. [28], which can be a source of confusion since the same nomenclature is used for the isoforms with respect to amino acid identity at position 7).

Surfactins are known to have antimicrobial properties that act against both bacteria and fungi [29]. They can insert into bacterial cell membranes, solubilizing the fluid phospholipid bilayer and creating pores and ion channels [29, 30]. Surfactins have also been shown to interfere with protein processing and secretion. For example, they disrupt aerial development of *Streptomyces* by interfering with the peptide SapB [31]. For antifungal activity, surfactins can inhibit glucan synthase, which is involved in cell wall synthesis, and can induce apoptotic markers [30]. In addition, surfactins are thought to play a key role in triggering ISR [32]. They may also contribute to swarming motility [33, 34], which can help achieve effective rhizosphere colonization and facilitate plant growth promoting traits while simultaneously inhibiting competing microorganisms via antimicrobial activity [34].

Despite the inability of either live *B. velezensis* 9D-6 or its surfactins to inhibit growth of *P. syringae* DC3000 in plate assays, *B. velezensis* 9D-6 proved effective against this phytopathogen in a plant system. It reduced both observable symptoms of *P. syringae* DC3000 in plants, as well as *P. syringae* DC3000 colonization of plant roots. These results indicate that *B. velezensis* 9D-6 produces a biocontrol effect attributable to something other than bacteriostatic or bactericidal compounds. Such biocontrol mechanisms could include adverse effects of sub-inhibitory concentrations of antimicrobials, which may possibly include surfactins. For example, secondary metabolites or enzymes can derail normal signaling pathways in other bacteria, including those leading to biofilm formation [31], which may be important to *P. syringae* DC3000’s survival or adhesion on plant roots but not on solid laboratory medium. Alternatively, *B. velezensis* 9D-6 may otherwise outcompete *P. syringae* DC3000 for resources that are more limiting in the plant system compared to the nutrient plate, or more limiting specifically within a biofilm, such as nutrients, oxygen, or space. This could include contact-mediated competition, where a membrane protein or secretion system delivers toxins to non-sibling cells in close proximity [31]. Alternatively, *B. velezensis* 9D-6 may promote ISR against *P. syringae* DC3000, a mechanism which may also involve surfactin [32] or butanediol, production of which by *B. velezensis* 9D-6 is inferred from the genome.

## Conclusions

Here, we showed that *B. velezensis* 9D-6 exhibits, overall, a high degree of inhibition against phylogenetically diverse microbial pathogens. Its genome contains a unique complement of 13 gene clusters that are expected to participate in antimicrobial production, with [Leu^7^] surfactins C_14_ and C_15_ confirmed as contributing at least to antibacterial activity in vitro. In addition, we found *B. velezensis* 9D-6 is able to reduce root colonization by *P. syringae* DC3000, whose in vitro growth was not inhibited, demonstrating that *B. velezensis* 9D-6 can use additional mechanisms to control phytopathogens. *B. velezensis* 9D-6 is therefore a candidate biopesticide. The data presented here not only highlight the potential of *B. velezensis* 9D-6 as a biocontrol agent against phytopathogens, but also warrant further research and understanding of *B. velezensis* 9D-6, and may therefore contribute to future utilization of this strain.

## Methods

### Isolation of *B. velezensis* 9D-6

Soil samples were collected from Blizman potato fields in Norfolk County, Ontario, Canada in the summer of 2012. Over the previous three years, bio-organic fertilizer was added to the soil each spring in an effort toward natural remediation. In 2012 (the fourth year), 10.0 g of moist soil was collected, placed in 95 mL of sterile water, and shaken for 10 min. Then, 1.0 mL of this suspension was transferred for serial dilution up to 10^− 10^, and the dilutions were plated on tryptic soy agar (TSA) for 48 h at 28 °C to attain single colonies. Permission for this research was obtained from the Canadian Food Inspection Agency (CFIA).

### In vitro antagonism assays against microbial pathogens

Six-hundred bacterial strains were further screened for their ability to suppress phytopathogens under in vitro conditions. Antimicrobial discs were prepared by inoculating 0.5 mm discs of P8 Filter Paper (Thermo Fisher Scientific, Pittsburgh, PA, USA) with 50 μL of 10^9^ CFU/mL *B. velezensis* 9D-6 suspended in 0.85% NdaCl. To test inhibition of pathogenic bacteria, 100 μL of each bacterial test strain at 10^9^ CFU/mL in 0.85% NaCl were spread onto separate LB agar plates, and an antimicrobial disc containing *B. velezensis* 9D-6 was placed in the centre of the plate. After two days incubation at 28 °C, zones of inhibition around the discs were recorded. Bacterial test strains were *Bacillus cereus*, *Clavibacter michiganensis*, *Erwinia amylovora*, *Pantoea agglomerans*, *Pseudomonas syringae* DC3000, *Ralstonia solanacearum*, *Xanthomonas campestris*, and *Xanthomonas euvesicatoria*.

To test inhibition of pathogenic eukaryotes, four plugs from each tested strain were evenly spaced at the peripheries of a potato dextrose agar plate, and an antimicrobial disc containing *B. velezensis* 9D-6 was placed in the centre of the plate. After one to two weeks of incubation (depending on the growth rate of the test strain) at room temperature, zones of inhibition around the discs were recorded. Eukaryotic test strains were *Alternaria solani*, *Cochliobolus carbonum*, *Fusarium oxysporum*, *Fusarium solani*, *Gibberella pulicaris*, *Gibberella zeae*, *Monilinia fructicola*, *Pyrenochaeta terrestris*, *Pythium mamillatum*, and *Rhizoctonia solani*.

### 16S rRNA sequencing and analysis

Bacterial genomic DNA was used for PCR amplification of 16S rRNA. For genomic DNA isolation, a single bacterial colony was inoculated into 2.5 ml of LB broth and grown for 16 h at 30 °C with shaking at 200 rpm. Cells were collected by centrifugation of 1.5 ml culture at 13,000 rpm for 5 min and bacterial genomic DNA was isolated using GenElute Bacterial Genomic DNA Isolation kit (Sigma-Aldrich Co., St. Louis, MO, USA) in accordance with the manufacturer’s protocol. PCR amplification of an approximately 1,500 base pair sequence of the bacterial 16S rDNA gene was performed with primers 8F (5′- AGAGTTTGATCCTGGCTCAG-3′) and 1492R (5’-GGTTACCTTGTTACGACTT-3′) [35]. Each 50 μL PCR mixture contained 1.5 units Phusion High-Fidelity DNA Polymerase (Thermo Fisher Scientific Inc., Waltham, MA, USA), 1X PCR buffer, 2.0 mM MgCl2, 200 μM dNTPs, 2.5 μM of each primer, 50 ng of genomic DNA template, and ultrapure water (Sigma-Aldrich Co.). The cycle parameters were as follows: initial denaturation at 95 °C for 5 min, followed by 30 cycles of denaturation at 94 °C for 30 s, annealing at 57 °C for 45 s, and extension at 72 °C for 60 s, with the final overall extension at 72 °C for 10 min. The 16S PCR products were purified by using QIAquick PCR Purification kit (Qiagen, Hilden, Germany) and sequenced with 8F and 1492R primers on a 3730 DNA Analyzer (Thermo Fisher Scientific Inc.) at Agriculture and Agri-Food Canada (London, Ontario, Canada). The 16S rRNA gene fragment was compared with the NCBI nucleotide database using Blastn to determine the closest taxonomic relatives.

### Phylogenetic tree construction

A phylogenetic tree was constructed based on RNA polymerase β subunit (*rpo*) gene sequences [9] obtained from the genome sequence (see below) to estimate the evolutionary position of *B. velezensis* 9D-6 relative to other *B. velezensis* strains. The Rpo protein sequence of *Bacillus subtilis subspecies subtilis* strain 168 (GenBank Accession NP_387988) was used as a query for translated BLAST (tblastn) against *B. velezensis* gene sequences in the NCBI database. In total, 28 *B. velezensis rpo* sequences were copied and aligned with *rpo* from *B. subtilis subtilis* 168 using WebPRANK software [36]. The type strains of *B. velezensis* are NRRL B-41580 and KCTC 13012 [37], but neither of their genome sequences are complete, and they were therefore excluded from the analysis. Phylogenetic trees were constructed using MrBayes v.3.2 [38] with the generalised time reversible (GTR) substitution model and default parameters for run length and frequencies. The *B. subtilis subtilis* 168 *rpo* was designated as an outgroup. Following analysis, the standard deviation of spilt frequencies was 0.006566 and the potential scale reduction factor (PSRF) was 1.000 or 1.001 for all factors. The majority-rule consensus tree was visualized using TreeGraph2 [39].

### Extraction and identification of antimicrobial compounds

To identify potential antimicrobial compounds produced by *B. velezensis* 9D-6, metabolites were isolated from a liquid LB culture grown at 28 °C for 72 h by ethyl acetate, liquid/liquid extraction. The extract was dried and resuspended in acetonitrile. After verifying the antibacterial and antifungal activity of the crude solution, compounds were separated into 22 fractions using a C18 reverse-phase high performance liquid chromatographer on a 1260 Infinity Series (Agilent Technologies, Santa Clara, CA, USA) eluted using an acetonitrile-water gradient with 0.1% formic acid.

Fractions were each tested in duplicate for their antibacterial activity against *C. michiganensis*. Each antimicrobial disc was prepared by adding 50 μL of a fraction to a 0.5 mm disc of P8 Filter Paper (Thermo Fisher Scientific, Pittsburgh, PA, USA), which was then placed onto an LB agar plate spread with 100 μL of 109 CFU/mL *C. michiganensis* in 0.85% NaCl. Controls used acetonitrile in place of the metabolite fraction.

Fractions demonstrating antibacterial effects were individually analyzed using ultraviolet-visible (UV-vis) spectroscopy at 210 nm and 254 nm to identify UV-active compounds. These compounds were isolated and tested in a final in vitro antibacterial plate assay against *C. michiganensis*. The compounds of bioactive fractions were characterized by high resolution LC-MS/MS using a Q-Exactive Quadrupole-Orbitrap Mass Spectrometry (Thermo Fisher Scientific, Waltham, MA, USA). Full MS and MS/MS spectra were analyzed manually with XCalibur software (Thermo Fisher Scientific, Waltham, MA, USA). The full MS data was used to identify possible molecular formula (< 3 ppm). Formulas were than searched against the AntiBase 2012 Natural Compound Identifier database [40] to identify purified compounds. When possible, the MS/MS spectra were used for de novo sequencing of peptide containing compounds to confirm possible Antibase identified compounds.

The surfactin fraction was then tested for activity against *P. syringae* DC3000 and *C. carbonum*. Discs were inoculated with 50 μL of the fraction at concentrations of 10 mg/mL, 5 mg/mL, 2.5 mg/mL, and 1 mg/mL. Discs were then placed onto LB agar plates that were either spread with *P. syringae* DC3000 or contained a central agar plug of *C. carbonum*.

### In vivo biocontrol

To determine whether alternative control mechanisms (such as induced systemic resistance) might be elicited by *B. velezensis* 9D-6, we tested its ability to reduce root colonization by *P. syringae* DC3000, a Gram-negative bacterium whose growth in vitro was not inhibited by *B. velezensis* 9D-6. *P. syringae* DC3000 was labeled using a red fluorescent protein (RFP) reporter gene construct based on the plasmid pME6010 [41, 42], and used to inoculate *Arabidopsis thaliana* (L.) Heynh seedlings in hydroponic medium (2.5 × 10^5^ CFU/mL), with or without *B. velezensis* 9D-6 (2.5 × 10^6^ CFU/mL), as previously described [43]. The *A. thaliana* (L.) Heynh seeds were obtained from the Arabidopsis Biological Resource Center (Columbus, OH, U.S.A) and grown as previously described [43].

After seven days, *A. thaliana* roots were removed from the medium, rinsed in ultrapure water to remove loosely bound material, and attachment by labeled *P. syringae* DC3000 was imaged using a DMIRE2 inverted microscope with confocal laser scanner (Leica Microsystems GmbH, Wetzlar, Germany). Samples were excited using a helium-neon 543/594 nm laser, and emission was detected at 590–630 nm under a 63 x water immersion objective with a numerical aperture of 1.4.

### Genome sequencing and annotation

Following isolation from potato field soil of Norfolk County, Ontario, Canada, a single colony of *B. velezensis* 9D-6 was grown for 48 h in 3 mL of lysogeny broth (LB) at 28 °C and 60 rpm in a TC-7 drum rotor (New Brunswick Scientific Co., Enfield, CT, USA). Genomic DNA was isolated from 1.5 mL of the culture using the GenElute Bacterial Genomic DNA kit (Sigma-Aldrich Co., St. Louis, MO, USA) according to the manufacturer’s protocol for Gram positive bacteria, with the exception that DNA was eluted in UltraPure DNase/RNase-free distilled water (Thermo Fisher Scientific Corp., Waltham, MA, USA). DNA quality was assessed by agarose gel electrophoresis.

Genomic DNA was sequenced at AGCT Inc. (Wheeling, IL, USA) using the MiSeq next generation sequencing platform (Illumina Inc., San Diego, CA, USA). Libraries were constructed using the NexteraXT DNA sample preparation kit (Illumina Inc., San Diego, CA, USA) with a target average insert size of 500–600 bp. Sequencing generated 5,453,964 raw read pairs of 2 × 150 bp read length on average. Adaptor sequences, low quality sequences, and short reads were filtered out using BaseSpace (Illumina Inc., San Diego, CA, USA), Trim Galore (Babraham Bioinformatics, Babraham, UK), and Sickle [44]. The remaining 4,722,973 trimmed read pairs were assembled de novo and scaffolded with SPAdes v. 3.50 [45] into 47 contigs with an N_50_ of 507 kb spanning 100x coverage.

The draft genome was aligned with test reference genomes, including that of *B. velezensis* G341 (GenBank Accession CP011686), using the Mauve Multiple Genome Alignment tool [46] version 2.4.0 to generate a preliminary chromosome map. Primers were designed based on the contig orientations suggested by the chromosomal map, and connections were confirmed by Sanger sequencing of PCR products followed by alignment with the draft genome using SeqMan Pro (DNASTAR Inc., Madison, WI, USA) [47]. All gaps were closed with one of two repeat sequences; one containing a transposon region and one containing an rDNA operon sequence. After closure and validation of gaps between draft contigs, the final completed genome was assembled into a single 3.96 Mb chromosome using the largest 20 contigs. The remaining 27 contigs were identified as redundant fragments.

Numbers of genes (rRNA genes, protein coding genes, pseudogenes) were predicted on the DOE-JGI Microbial Genome Annotation Pipeline (MGAP v.4) [48]. In silico DNA-DNA hybridization (*is*DDH) was performed via the GGDC web server (http://ggdc.gbdp.org) using formula 2 [49]. Secondary metabolite clusters were identified using both MGAP v.4 and antiSMASH3.0 [50]. The genome was also mined in silico using target amino acid sequences from closely related species from the UniProtKB sequence database [51] and comparing them to translated draft genome nucleotide data using tBLASTn.

## Additional files

Additional file 1: Growth of *B. velezensis* 9D-6 in liquid LB at various temperatures. (DOCX 16 kb)

Additional file 2: Growth of *B. velezensis* 9D-6 in liquid LB at various pH. (DOCX 15 kb)

Additional file 3: Genome summary. (DOCX 16 kb)

Additional file 4: Number of genes associated with general COG functional categories. (DOCX 16 kb)

## Abbreviations

Bt: *Bacillus thuringiensis*
CFU: colony forming unit
GTR: generalised time reversible model of nucleic acid substitution
ISR: induced systemic resistance
nrps: nonribosomal peptide synthetase
PGPB: plant growth promoting bacterium
PSRF: potential scale reduction factor
RFP: red fluorescent protein
t2pks: Type II polyketide synthase
t3pks: Type III polyketide synthase
Tat: twin-arginine translocation
transatpks: trans-acyltransferase modular polyketide synthase
