# Supplementary material for: Characterization and complete genome analysis of the surfactin-producing, plant-protecting bacterium Bacillus velezensis 9D-6
Source: BMC Microbiol. 2019 Jan 8;19:5. doi: 10.1186/s12866-018-1380-8 (PMC6325804; doi:10.1186/s12866-018-1380-8)
Supplement: Supplementary file 1 — Growth of B. velezensis 9D-6 in liquid LB at various temperatures. (DOCX 16 kb) [file 12866_2018_1380_MOESM1_ESM.docx]

**Additional file 1**: Growth of *B. velezensis* 9D-6 in liquid LB at various temperatures.

| **Temp.** | **OD_600_ at 24 h.** | **Fold increase*** |
| --- | --- | --- |
| 12ºC | 1.38 | 15x |
| 16 ºC | 1.32 | 14x |
| 30 ºC | 3.58 | 38x |
| 37 ºC | 2.98 | 32x |
| 45 ºC | 2.18 | 23x |
| 50 ºC | 1.23 | 13x |

*Starting OD_600_ at 0 h. was 0.093.
