# Supplementary material for: Characterization and complete genome analysis of the surfactin-producing, plant-protecting bacterium Bacillus velezensis 9D-6
Source: BMC Microbiol. 2019 Jan 8;19:5. doi: 10.1186/s12866-018-1380-8 (PMC6325804; doi:10.1186/s12866-018-1380-8)
Supplement: Supplementary file 2 — Growth of B. velezensis 9D-6 in liquid LB at various pH. (DOCX 15 kb) [file 12866_2018_1380_MOESM2_ESM.docx]

**Additional file 2**: Growth of *B. velezensis* 9D-6 in liquid LB at various pH.

| **pH** | **OD_600_ at 12 h.** | **Fold increase*** | **OD_600_ at 24 h.** | **Fold increase*** |
| --- | --- | --- | --- | --- |
| 4 | 0.03 | 2x | 0.03 | 2x |
| 5 | 0.12 | 8x | 0.24 | 16x |
| 6 | 0.23 | 15x | 0.35 | 23x |
| 7 | 0.26 | 17x | 0.48 | 32x |
| 8 | 0.20 | 13x | 0.30 | 20x |
| 9 | 0.00 | 0x | 0.19 | 13x |

*Starting OD_600_ at 0 h. was 0.015.
