# Supplementary material for: Characterization and complete genome analysis of the surfactin-producing, plant-protecting bacterium Bacillus velezensis 9D-6
Source: BMC Microbiol. 2019 Jan 8;19:5. doi: 10.1186/s12866-018-1380-8 (PMC6325804; doi:10.1186/s12866-018-1380-8)
Supplement: Supplementary file 3 — Genome summary. (DOCX 16 kb) [file 12866_2018_1380_MOESM3_ESM.docx]

**Additional file 3**: Genome summary.

| **Attribute** | **Value** | **% of Total** |
| --- | --- | --- |
| Genome size (bp) | 3,963,726 | 100.0 |
| DNA coding (bp) | 3,565,928 | 90.0 |
| DNA G+C (bp) | 1,838,113 | 46.4 |
| DNA scaffolds | 1 |  |
| Total genes | 3,942 |  |
| Protein coding genes | 3,849 | 97.6 |
| RNA genes | 93 | 2.4 |
| Pseudo genes | 82 | 2.1 |
| Genes in internal clusters | 987 | 25.0 |
| Genes with function prediction | 3,164 | 80.3 |
| Genes assigned to COGs | 2,736 | 69.4 |
| Genes with Pfam domains | 3,339 | 84.7 |
| Genes with signal peptides | 191 | 4.9 |
| Genes with transmembrane helices | 1,026 | 26.0 |
| CRISPR repeats | 0 | 0.0 |
