# Supplementary material for: Characterization and complete genome analysis of the surfactin-producing, plant-protecting bacterium Bacillus velezensis 9D-6
Source: BMC Microbiol. 2019 Jan 8;19:5. doi: 10.1186/s12866-018-1380-8 (PMC6325804; doi:10.1186/s12866-018-1380-8)
Supplement: Supplementary file 4 — Number of genes associated with general COG functional categories. (DOCX 16 kb) [file 12866_2018_1380_MOESM4_ESM.docx]

**Additional file 4:** Number of genes associated with general COG functional categories.

| **Code** | **Value** | **%age** | **Description** |
| --- | --- | --- | --- |
| J | 210 | 5.5 | Translation, ribosomal structure and biogenesis |
| A | 0 | 0.0 | RNA processing and modification |
| K | 261 | 6.8 | Transcription |
| L | 109 | 2.8 | Replication, recombination and repair |
| B | 1 | 0.0 | Chromatin structure and dynamics |
| D | 54 | 1.4 | Cell cycle control, Cell division, chromosome partitioning |
| V | 80 | 2.1 | Defense mechanisms |
| T | 157 | 4.1 | Signal transduction mechanisms |
| M | 178 | 4.6 | Cell wall/membrane biogenesis |
| N | 57 | 1.5 | Cell motility |
| U | 27 | 0.7 | Intracellular trafficking and secretion |
| O | 112 | 2.9 | Posttranslational modification, protein turnover, chaperones |
| C | 167 | 4.3 | Energy production and conversion |
| G | 238 | 6.2 | Carbohydrate transport and metabolism |
| E | 289 | 7.5 | Amino acid transport and metabolism |
| F | 90 | 2.3 | Nucleotide transport and metabolism |
| H | 180 | 4.7 | Coenzyme transport and metabolism |
| I | 141 | 3.7 | Lipid transport and metabolism |
| P | 165 | 4.3 | Inorganic ion transport and metabolism |
| Q | 103 | 2.7 | Secondary metabolites biosynthesis, transport and catabolism |
| R | 259 | 6.7 | General function prediction only |
| S | 195 | 5.1 | Function unknown |
| - | 1206 | 31.3 | Not in COGs |

The total is based on the total number of protein coding genes in the genome.
